# Standardized Chinese Formula Xin-Ke-Shu inhibits the myocardium Ca^2+^ overloading and metabolic alternations in isoproterenol-induced myocardial infarction rats

**DOI:** 10.1038/srep30208

**Published:** 2016-07-26

**Authors:** Yue-Tao Liu, Chao Zhou, Hong-Mei Jia, Xing Chang, Zhong-Mei Zou

**Affiliations:** 1 Institute of Medicinal Plant Development, Chinese Academy of Medical Sciences and Peking Union Medical College, Beijing 100193, P. R. China; 2 Modern Research Center for Traditional Chinese Medicine of Shanxi University, NO. 92, Wucheng Road, Taiyuan 030006, Shanxi, P. R. China

## Abstract

Xin-Ke-Shu (XKS) is a traditional Chinese patent medicine used for treatment of coronary heart diseases in China. However, its mechanism of action is still unclear. In this paper, the mediation of XKS on the isoproterenol (ISO)-induced myocardial infarction (MI) rat were evaluated based on a tissue-targeted metabonomics *in vitro/vivo*. The result indicated that twelve metabolic pathways were involved in the therapeutic effect of XKS *in vivo*, where seven pathways were associated with the Ca^2+^ overloading mechanism. In agreement with regulation on metabolic variations, XKS markedly reversed the over-expressions of three involved proteins including phospholipase A2 IIA (PLA2 IIA), calcium/calmodulin-dependent protein kinase II (CaMK II) and Pro-Caspase-3. The metabolic regulations of XKS on H9c2 cell also partially confirmed its metabolic effect. These metabolic characteristics *in vitro/vivo* and western blotting analysis suggested that XKS protected from MI metabolic perturbation major *via* inhibition of Ca^2+^ overloading mechanism. Furthermore, 11 active ingredients of XKS exerted steady affinity with the three proteins through the molecular docking study. Our findings indicate that the metabonomics *in vitro/vivo* combined with western blotting analysis offers the opportunity to gain insight into the comprehensive efficacy of TCMs on the whole metabolic network.

Coronary heart disease (CHD) has a high prevalence in developing and developed countries, and myocardial infarction (MI) accounts for majority of deaths and disabilities^1^. MI is the acute condition of myocardial necrosis that occurs as a result of imbalance between coronary blood supply and myocardial demand^2^. A great deal of evidences have ascribed MI to specific cell ultra pathways, including an increase in free radicals formation, lipid peroxidation, cardiomyocyte apoptosis, interference of calcium dynamics, abnormalities in the mitochondria, alteration of cardiac energetic, irreversible damage of DNA and other putative mechanisms^3^,^4^. As the risk factors are complicated, most modern western medicines are based on the lock-and-key theory, which attempts to use one single agent to hit one target in order to regulate CHD, and usually fails to fight multi-risk diseases. Recently, combination therapy (multi-component medicine such as the polypill) has gained widespread acceptance^5^.

Traditional Chinese medicine (TCM) formulae are natural multi-component medicines and pursue a holistic therapeutic effect with few side effects^6^. Xin-Ke-Shu (XKS), a traditional Chinese patent medicine consisting of five commonly used Chinese herbs: i.e the roots of *Salviae miltiorrhizae* Bge. (Dan-Shen), the roots of *Pueraria lobata* (Willd.) Ohwi. (Ge-Gen), the roots of *Panax notoginseng* (Burk.) F. H. Chen. (San-Qi), the fruit of *Crataegus pinnatifide* Bge. (Shan-Zha), and the roots of *Aucklandia lappa* Decne (Mu-Xiang), has been widely used for the treatment of myocardial ischemia and reperfusion injury^7^,^8^. The chemical constituents in XKS preparation are qualitatively and quantitatively investigated by an optimized LC-LTQ-Orbitrap method^9^,^10^. Our previous study has demonstrated that the effect of XKS on atherosclerotic myocardial ischemia rabbit attributes to the inhibition of the coronary stenosis and increase of eNOS and VCAM-1 expression^11^. Additionally, pretreatment of XKS significantly inhibits the activities of plasma enzymes (creatinenases, lactate dehydrogenase and aspartate transaminase), and ameliorates neutrophil recruitment and endothelial injury in a MI rat model induced by isoproterenol (ISO)^12^. This highlight links the coronary arteriole spasm and XKS protection, and reveals a systemic mechanism exploiting from metabolites of soluble epoxide hydrolase. Therefore, investigation into the altered metabolome is required for deeply understanding the molecular mechanism of XKS against MI.

Metabonomics, as an important platform of systems biology, holds promise for the discovery of pathways linked to disease processes and pharmacological action of drugs^13^,^14^. In agreement with the holistic thinking of TCM, metabonomics has shown potential in evaluation of the therapeutic effect of TCMs, and may provide the links needed for the complex metabolite mixtures in TCMs and molecular pharmacology^15^,^16^. In our previous study based on plasma metabonomics, pretreatment of XKS regulates fifteen pathological biomarkers and two pharmaco biomarkers to protect the MI metabolic perturbations, major involving into lipid pathways, amino acid metabolism and purine metabolism^12^. Compared with plasma, ischemia myocardium tissue can offer a unique perspective on localized information related to MI^17^. So it is necessary to investigate the mechanism of actions of XKS on the targeted-tissue, in order to illustrate its underlying metabolic response to the metabolic alternations in ischemia tissue. Specifically, the further evaluation based on cell directly derived from corresponding tissue, could imitate and characterize the local metabolic alternations of the related organs, which might be helpful to understand the regulative mechanism of XKS on the microscopic level.

Here, an integrated approach utilizing UPLC-Q/TOF MS and ^1^H NMR together for a comprehensive metabonomics was applied to investigate the tissue-specific metabolic regulation of XKS against ISO-induced MI rat. Meanwhile, the detailed molecular mechanism related with Ca^2+^ overloading was also explored to understand the therapeutic effect of XKS against MI. At last, the regulation of XKS towards to H9c2 cell metabolic disturbances was used to confirm and improve the knowledge about the progression of MI and the therapeutic basis of XKS, which coupled with the molecular docking study. To the best of our knowledge, this is the first report about the inhibited Ca^2+^ overloading mechanism of TCM protecting against MI based on a tissue-targeted metabonomics *in vivo* and *in vitro*.

## Results

### Biochemistry assays and Pathological analysis

As described in our published work^12^, rats treated with ISO showed significant increase in the levels of CK (creatinenases), LDH (lactate dehydrogenase), AST (aspartate transaminase) and MDA (malondialdehyde) and decreases SOD (superoxide dismutase) compared with normal rats (control group), indicating ISO successfully induced acute MI. Oral administration with XKS could significantly ameliorate these abnormities (*p* <  0.05 or *p* <  0.01). Meanwhile, treatment of XKS showed a moderated markedly effect on pathological changes in model rats, including widen sarcoplasm, ghost cell, stainless area, red blood cells out of vessels, and infiltrated neutrophilic granulocytes.

### Myocardial metabolic profiles of the MI rats associated with XKS pretreatment

**Figure 1:**
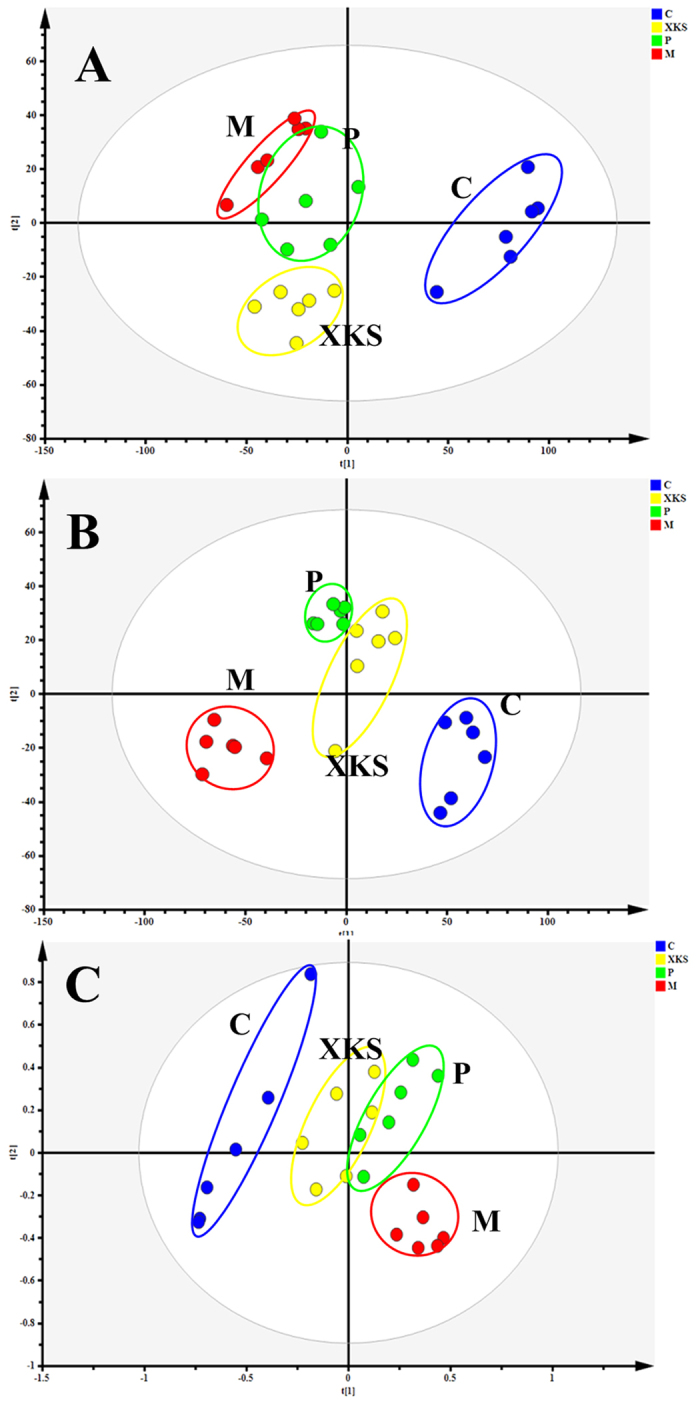
PCA score plots of myocardium samples collected from different treatment groups of rats based on UPLC-Q/TOF MS in positive mode ((**A**) *R*^*2*^X = 0.766, *Q*^*2*^ (cum) = 0.452) and in negative mode ((**B**) *R*^*2*^X = 0.531, *Q*^*2*^ (cum) = 0.709); PCA score plots of myocardium samples collected from different treatment groups of rats based on ^1^H NMR (C, *R*^*2*^X = 0.955, *Q*^*2*^ (cum) = 0.746). (**C**) Control Group, (M), Model Group, (P), Positive Group, (XKS) XKS Group.

The myocardial tissues collected from each group were analyzed by UPLC-Q/TOF MS and ^1^H NMR, respectively. PCA analyses of UPLC-Q/TOF MS and ^1^H NMR data indicated that the metabolic profiles of rats in the model group deviated from the control, suggesting that significant biochemical changes were induced by ISO (Fig. 1). The metabolic profiles of rats in XKS treated group fairly differed from the MI group and closed to the control, indicating the abnormities induced by MI were significantly improved after pretreatment of XKS. Meanwhile, XKS treated group was much closer to control group than propranolol treated group, which was consistent with our histopathological observation^12^.

### Regulation of XKS on the potential MI biomarkers

OPLS-DA analysis for the variables contributing to the classification between the control and MI groups was carried out based on the UPLC-Q/TOF MS and ^1^H NMR data of myocardium samples. A clear separation was observed between them in score plot (Figure S1), indicating that some endogenous metabolites were greatly perturbed by ISO-induced MI. Variables with high VIP (Table S2) contributing significantly to the clustering were considered as potential biomarkers related to the MI. A total of twenty-two potential biomarkers related to MI were identified from variables in S-plots of OPLS-DA. Among them, eleven (**1**–**11**) were detected from UPLC-Q/TOF MS analysis and twelve (**7**, **12**–**22**) from ^1^H NMR data (as showed in Table S1).

**Figure 2:**
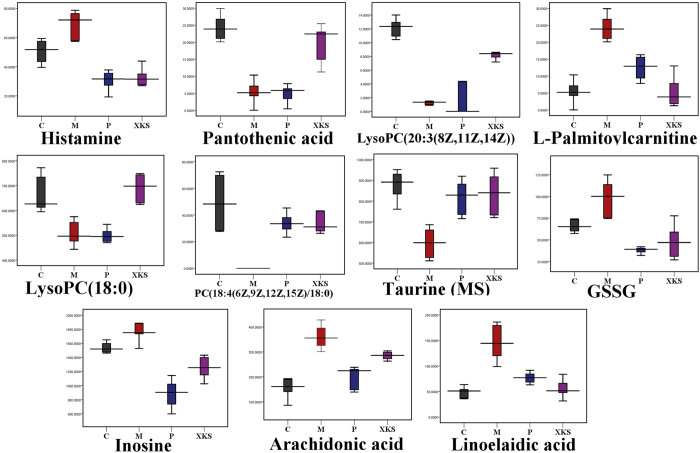
Box plots showing integrated intensities for 11 selected LC-MS-indentified biomarkers reversed by XKS in control, model, positive and XKS pretreatment groups. (C), Control Group, (M), Model Group, (P), Positive Group, (XKS), XKS Group.

**Figure 3:**
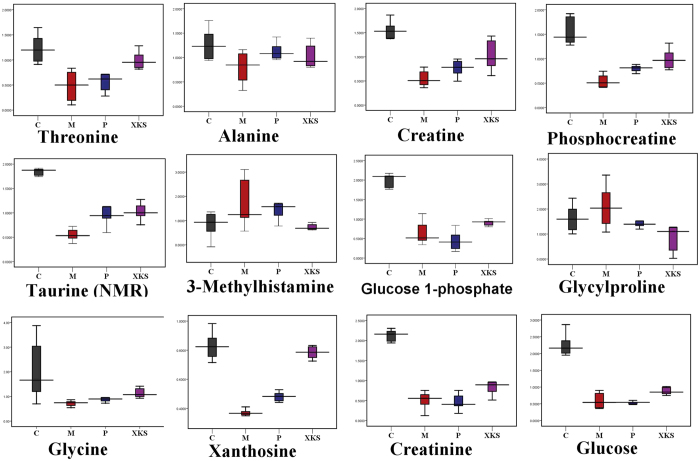
Box plots showing integrated intensities for 11 selected NMR-indentified biomarkers reversed by XKS in control, model, positive and XKS pretreatment groups. (C), Control Group, (M), Model Group, (P), Positive Group, (XKS), XKS Group.

With administration of XKS, the increased levels of histamine (**1**), L-palmitoylcarnitine (**4**), GSSG (**8**), inosine (**9**), arachidonic acid (**10**), linoelaidic acid (**11**), 3-methylhistamine (**16**) and glycylproline (**18**) in the myocardial tissue of ISO-induced MI rats were down-regulated. Meanwhile, the decreased levels of pantothenic acid (**2**), lysoPC(20:3 (8Z, 11Z, 14Z)) (**3**), lysoPC(18:0) (**5**), PC(18:4 (6Z, 9Z, 12Z, 15Z)/18:0) (**6**), taurine (**7**), threonine (**12**), alanine (**13**), creatine (**14**), phosphocreatine (**15**), glucose 1-phosphate (**17**), glycine (**19**), xanthosine (**20**), creatinine (**21**) and glucose (**22**)induced by ISO were significantly medicated by pretreatment of XKS. However, propranolol did not show any effects on metabolites **2**, **3**, **5**, **13**, **16**, **17**, **21** and **22** (Figs 2 and 3).

### Regulation of XKS on the related metabolic network associated with the Ca^2+^ overloading mechanism in ISO-induced MI rats

Based on the identified potential biomarkers of ISO-induced MI in the myocardium tissues of rats and the mediations of XKS, the affected metabolic network related to the myocardium protection of XKS was depicted according to the Kyoto Encyclopedia of Genes and Genomes (KEGG) (http://www.genome.ad.jp/kegg/). The results supported our previous research^12^, where inflammation, oxidative stress, as well as alterations of energy metabolism were involved into the protection of XKS against MI. The results also provided new clues for the myocardium protection mechanism of XKS, which XKS could regulate those metabolic disturbances related Ca^2+^ overloading mechanism in ISO-induced MI rats.

XKS mediated the deviation of 22 potential biomarkers related to MI, which involved 12 perturbed metabolic pathways according to KEGG, including glycerophospholipid metabolism (**3**, **5** and **6**), arachidonic acid metabolism (**10** and **11**), fatty acid *β*-oxidation pathway (**4** and **11**), taurine and hypotaurine metabolism (**7**), glutathione metabolism (**8**), purine metabolism (**9** and **20**), collagen metabolism (**18**), histidine metabolism (**1** and **16**), pantothenate and CoA biosynthesis (**2**), glycine, serine and threonine metabolism (**12**, **13** and **19**), arginine and proline metabolism (**14**, **15** and **21**) and glycolysis (**17** and **22**). Among them, the first seven metabolic pathways were associated with the Ca^2+^ overloading mechanism.

### Glycerophospholipid metabolism

LysoPC (18:0) (**3**), PC (18:4(6Z,9Z,12Z,15Z)/18:0) (**5**) and lysoPC (20:3(8Z,11Z,14Z)) (**6**), belonging to glycerophospholipids, are important constituents of cell membranes including endothelial and myocardial cells^18^. Overloading of the intracellular Ca^2+^ can activate the phospholipase in cell membrane, which promotes the cell membrane disruption and further results in the decrease of lysoPCs generation^19^. As a result, the decreased levels of **3**, **5** and **6** were observed in rat myocardium with administration of high dose ISO. Their abnormalities could be inhibited by XKS, while propranolol showed no amelioration. The results suggested that the protective effect of cell membrane might contribute to the antagonism of XKS against MI.

### Arachidonic acid metabolism

Arachidonic acid (**10**), one polyunsaturated and essential fatty acid, could mediate inflammation and the functioning of several organs and systems. Especially, arachidonic acid (**10**) is oxygenated and further transformed into a variety of products which mediate or modulate inflammatory reactions^20^. PLA2 IIA, a calcium-independent phospholipase A2, was identified to be a mediator of systemic inflammation and risk of MI^21^,^22^. Its activation could result in the increasing of arachidonic acid (**10**)^19^. In this study, the elevated level of **10** was observed, which resulted in the release of inflammatory active substances, such as serotonin, histamine, prostaglandins, cytokines and bradykinins. XKS and propranolol treatments could regulate this abnormality, which suggested that XKS could protect the injury of myocardium from the occurrence of inflammation.

### Fatty acid *β*-oxidation pathway

Fatty acids are the largest energy reserve by *β*-oxidation in the body, which have been considered as independently predictive of future cardiovascular events^23^. The Ca^2+^ overloading induced by excessive ISO could activate the multifunctional CaMK II, which could promotes mitochondrial permeability transition pore (mPTP) opening and myocardial death, mitochondrial disruption and programmed cell death in response to ISO^24^, which result in the inhibition of fatty acid *β*-oxidation pathway and the disturbance of energy supply^25^,^26^. In MI condition, the *β*-oxidation of fatty acid would be inhibited, resulted in the accumulation of toxic intermediates such as L-palmitoylcarnitine (**4**)^27^. The increase of L-palmitoylcarnitine (**3**) in MI group was observed. Moreover, the increased levels of linoelaidic acid (**11**) (a derivative of an unsaturated fatty acid linoleic acid) was detected, which indicated that the oxidation of fatty acid was also inhibited in myocardium^28^. Regulation of **3** and **11** by XKS pretreatment indicated XKS could recover the dysfunction of mitochondrial oxidation in ISO-induced MI rats.

### Taurine and hypotaurine metabolism

Taurine (**6**) is a sulfur amino acid presenting in myocardial cells with high abundance^29^ and has exerts protective actions against MI through reducing Ca^2+^ overload by means of modulations of ion channels and exchange systems^30^. Additionally, taurine protects membrane lipids from peroxidation caused by free radicals^31^. In this work, taurine was significantly consumed by myocardial cells, suggesting the cells altered their metabolism while trying to reduce Ca^2+^ overload and maintain the function of myocardium. XKS and propranolol were observed to prevent the decreased tendency of taurine (**6**) in MI process, indicating therapeutic effects of XKS and propranolol may base on the modulations of Ca^2+^ overload and oxidant defense system to maintain taurine and hypotaurine metabolism.

### Glutathione metabolism

In the cellular defense system, GSH (reduced glutathione) plays an important protective role in the lipid peroxidation of the mitochondrial membrane^32^. During MI, the activation of CaMK II could promote the generation of oxygen free radicals, which contribute to myocardial tissue injury^33^. Low level of GSH was observed during increased oxidative stress and disturbance of Ca^2+^ influx induced by oxidative injury. It has been reported that isoproterenol could significantly deplete the levels of GSH, which could lead to accumulation of GSSG (**7**, oxidized glutathione)^34^. This report supports our findings that we observed an increase in GSSG levels in MI injured rats. Pretreatment of XKS and propranolol may maintain the concentration of GSSG at near normal levels through significantly increasing SOD activity and decreasing the Ca^2+^ influx.

### Purine metabolism

Under MI, hypoxanthine accumulates and is further metabolized to xanthine and urate by xanthine dehydrogenase or xanthine oxidase and converted to inosine^19^. During this process, the conversion of xanthine oxidase from xanthine dehydrogenase was activated by CaMK II^35^. It has been considered that xanthine oxidase could generate excess oxygen-free radicals and increasingly release of inosine (**8**), then caused ischemia injury in the pathway^36^. Significantly increase of inosine (**8**) was observed in present study, which has been reported as a marker of ischemic myocardium in urinary metabonomic study^37^. The decreased xanthosine (**20**) detected by NMR were also involved in purine metabolism, which was an important process in myocardial ischemia injury^38^. The reason for its reduction in myocardium of MI rat is probably related to the degradation of ATP, of which the elevated biosynthesis from biomolecules, such as xanthosine and lead to xanthosine decreasing in MI group. The pretreatment with XKS and propranolol could effectively ameliorate the abnormal change of these two biomarkers, suggested that the cardioprotective effect of XKS and propranolol partially attributed to the regulation of purine metabolism.

### Collagen metabolism

During MI, CaMK II could advance the activation of collagenase and the degradation of collagen^39^. Disturbance of collagen metabolism has been characterized that induce remodeling of the myocardium and ultimately deteriorate left ventricular (LV) function and facilitate the development of MI^40^. Glycylproline (**18**), the end product of collagen metabolism, were increased in MI rats in this study, meaning predominance of degradation over synthesis of collagen leads to the disruption and loss of myocardial collagen scaffold and/or decline in matrix tensile strength that can be responsible for ventricular dilatation and systolic dysfunction. Down-regulation of glycylproline (**18**) after XKS and propranolol treatments suggested that both of them had the potential to inhibit myocardial remodeling in the pathological process of MI.

In addition, to understand which pathways were the targeted pathways of XKS against MI, a comprehensive metabolic network to illustrate the pharmacological action of XKS was mapped (Figure S2) on MetaboAnalyst 2.0 (MetPA, http://www.metaboanalyst.ca/MetaboAnalyst/)^41^,^42^. The impact value of pathways on MetPA analysis was applied to evaluate the importance of the pathways on the efficacy of XKS (Table S2). Five disturbed metabolic pathways with Impact >0.1 were considered as the most relevant pathways to the action of XKS against MI^43^. They are taurine and hypotaurine metabolism, glycolysis, arachidonic acid metabolism, glycine, serine and threonine metabolism and histidine metabolism, respectively. Among them, two metabolic pathways, taurine and hypotaurine metabolism and arachidonic acid metabolism, were both involving in the Ca^2+^ overloading mechanism. Histamine (**1**), threonine (**12**), 3-methylhistamine (**16**) and glycine (**18**) provide substrates for energy metabolism, while glucose-1-phosphate (**17**) and glucose (**22**) are participated in glycolysis pathway. All of them were significantly altered, suggesting the deficiency of ATP biosynthesis under MI condition. XKS also exerted the potential protection on them to maintain the energy requirement. And these metabolites have been proved involving into the Ca^2+^ overloading mechanism and energy metabolism in the formation and development of MI^44^. In our study, the MetPA analysis based on metabonomics suggested that pretreatment of XKS could effectively protect from MI *via* its improvement of the Ca^2+^ overloading-related metabolic pathways, which provide one of the most influencing target related to the therapeutic intervention of XKS against MI.

### Effect of XKS on the expressions of PLA2 IIA, CaMK II and Pro-Caspase-3

**Figure 4:**
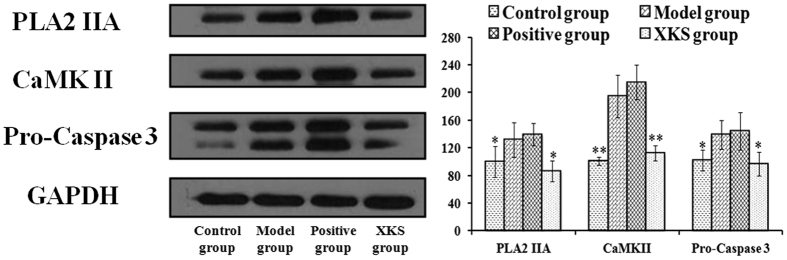
(**A**) XKS effect on PLA2 IIA, CaMK II and Pro-Caspase-3 in myocardium sample of the tested rats. (**B**) Bar graphs represent mean ± SD. of the expressions of PLA2 IIA, CaMK II and Pro-Caspase-3 in myocardium samples of the tested rats. ^a^Values are significant difference compared with control group, *p* < 0.05. ^b^Values are significant difference compared with control group, *p* < 0.01. ^c^Values are significant difference compared with model group, *p* < 0.05; ^d^Values are significant difference compared with model group, *p* < 0.01.

Under MI, Ca^2+^ overloading mechanism was currently considered as the final pathway of cell injury and death, which could initiate the activation of PLA2 IIA, CaMK II, etc. and further lead to the formation of MI. Moreover, it could indirectly trigger apoptosis induced by Caspase-3, of which the activation also depends on the participation of cytosolic free calcium^45^. The metabonomic results suggested that the protection of XKS from the perturbed metabolic alternations was primarily ascribed to the regulation of the Ca^2+^ overloading-related metabolic pathways. Thus, three proteins related to Ca^2+^ overloading mechanism, PLA2 IIA, CaMK II and Pro-caspase-3, were selected to evaluate the inhibition of XKS on the Ca^2+^ overloading mechanism, which might provide the linking between the upstream protein expression and the downstream endogenous metabolites in molecular pharmacology. The levels of them in all experimental groups were determined by the western blotting. The typical bands of expressions of them were illustrated in Fig. 4. The results showed that the expressions of PLA2 IIA, CaMK II and Pro-Caspase-3 in model group were significantly up-regulated compared with the control group (n = 3, *p* < 0.05 or *p* < 0.01). Pretreatment with XKS markedly reversed the over-expressions of these three proteins (*p* < 0.05). However, treatment with propranolol had no regulation on these three proteins (*p* > 0.05).

### Molecular docking analysis

**Figure 5:**
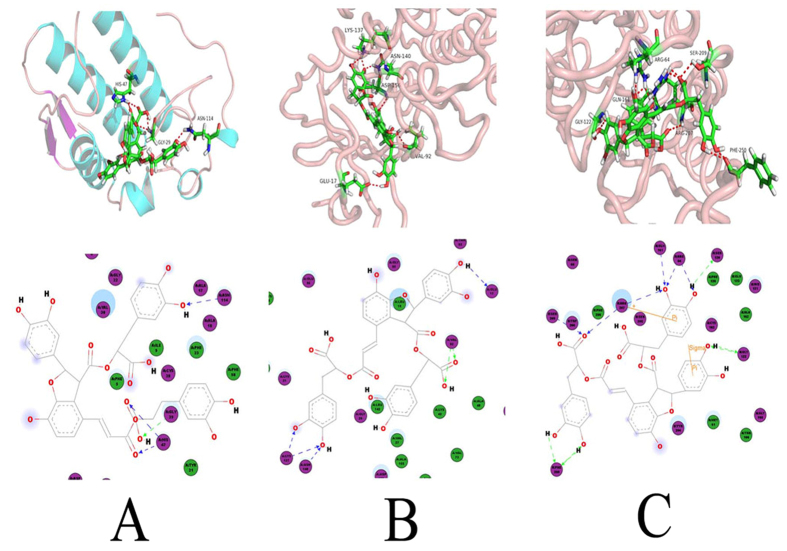
Computer-aided molecular docking. (**A**) The 3D and 2D map of predicted binding orientation of salvianolic acid B within PLA2 IIA active site. (**B**) The 3D and 2D map of predicted binding orientation of salvianolic acid B within CaMK II α active site. (**C**) The 3D and 2D map of predicted binding orientation of salvianolic acid B within Pro-Caspase3 active site.

Molecular docking of the 51 reported compounds^12^ was carried out with the aim of understanding the possible binding orientations within the PLA2 IIA, CaMK IIα and Pro-Caspase-3 active sites. The five highest ranked compounds within the tested three protein sites were illustrated in Table S7, respectively. 11 compounds, including salvianolic acid B, puerarin 7-*O*-glucoside, pueroside B, ginsenoside Rb1, ginsenoside Rg3, notoginsenosideK/gypenoside XVII, salvianolic acid C, lithosperimic acid, ginsenoside Rd, genistein-8-C-apiosyl(1-6)-glucoside and notoginsenoside R1, were able to connect steadily with PLA2 IIA, CaMK IIα and Pro-Caspase-3. Among them, salvianolic acid B showed the best bond within the substrate cavity of all the enzymes through our docking experiment. Therefore, these molecules could be viewed as promising compounds for the inhibition of Ca^2+^ overloading mechanism (Fig. 5).

### Effect of XKS on ISO-induced hypertrophy in H9c2 cells

The effects of XKS against ISO-induced hypertrophy on H9c2 cells were evaluated by MTT assay. The result indicated that XKS at 0.0625, 0.125 and 0.25 mg/mL could significantly inhibit the hypertrophy in a dose-depended manner (Figure S3). Especially, XKS can almost 100% inhibit the hypertrophy at 0.25 mg/mL. So this dose was chosen for the followed cell metabonomic study.

### Regulation of XKS on the related metabolic profile associated with ISO-induced hypertrophy in H9c2 cell

As showed in Figure S4, clear separation of the three groups was observed from the PCA analysis. The model presents satisfied classification ability among the experimental groups. Among them, control and model group could be classified clearly, indicating that there was significant metabolic difference between the two groups. The metabolic profile of XKS treated group fairly differed from the model group, which were closed to the control, indicating the deviations induced by ISO were significantly improved after pretreatment of XKS. The score plot of further OPLS-DA showed metabolic profiles of the control and model group were separated clearly (Figure S5). The metabolites contributed to the observed separation in the respective S-plot were selected as potential markers related to hypertrophy. As a result, ten metabolites, including dodecanoic acid, phytosphingosine, sphinganine, unknown (**C4**), lysoPC(18:2), dihydroceramide, lysoPC(20:4), PC(16:0/0:0), lysoPC(18:1) and cer(d18:0/26:0), were selected and identified as potential biomarkers related to ISO-induced hypertrophy in H9c2 cell, which were involved into glycerophospholipid metabolism, sphingolipid metabolism and fatty acid *β*-oxidation pathway. In cell hypertrophy group, nine metabolites except unknown (**C4**) were increased compared with control group. After XKS pretreatment, all the 10 metabolites were regulated significantly (Figure S6, *p* < 0.05 or *p* < 0.01).

## Discussion

**Figure 6:**
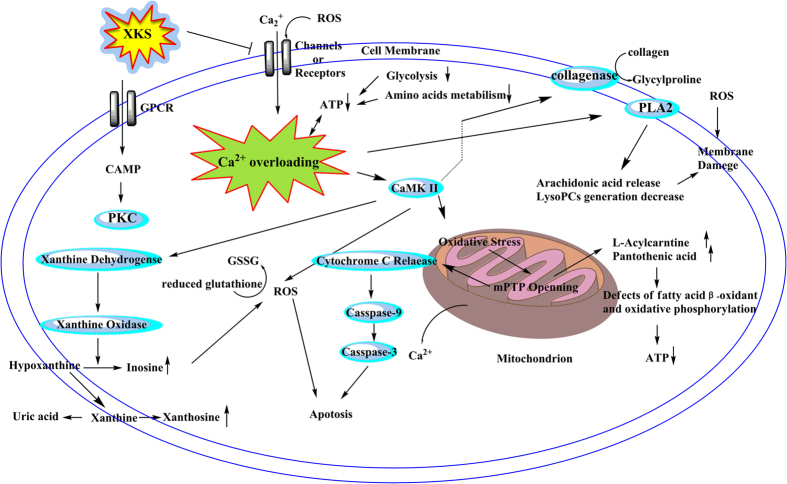
Effect of XKS on the related metabolic network associated with the Ca^2+^ overloading mechanism in ISO-induced MI rats.

Clinical and pharmacological basic researches have revealed that XKS pursues an overall therapeutic effect by means of hitting multiple targets with multiple components^11^. The plasma clinic parameters and pathology analysis suggest that pretreatment of XKS could effectively improve the myocardial injury induced by ISO. In our previous plasma metabonomic study, five involved metabolic pathways, glycerophospholipid metabolism, arachidonic acid metabolism, fatty acid *β*-oxidation pathway and purine metabolism, are related to the disorders of energy metabolism, inflammation and oxidation stress, which exerted indirect links with the development of the Ca^2+^ overloading mechanism in MI. However, these evidences are not enough to elucidate the development of Ca^2+^ overloading mechanism. Compared with plasma metabonomics, the tissue-targeted metabonomics could reveal the located and specific metabolic alteration in MI myocardium^17^. Thus, 22 myocardium biomarkers were identified to characterize the metabolic changes of MI and the pharmacodynamic efficacy of MI in the present study. Only L-palmitoylcarnitine and lysoPC (18:0) have been found in our plasma metabonomics. Both plasma and the tissue-targeted metabonomics suggested that lipid pathways were the targeted interventions of XKS against MI. Different from plasma metabonomics, significant alternations in myocardium metabolic profile were involved into the metabolism of saccharides, amino acids and nucleotides. Taurine was the key biomarker of XKS acting on MI myocardium with the highest Impact according to MetPA analysis, which provided a special clue to link the Ca^2+^ overloading mechanism with the pathogenesis of MI. Furthermore, the mediation of XKS on fatty acid *β*-oxidation pathway, arachidonic acid metabolism, glycerophospholipid metabolism, glutathione metabolism, purine metabolism and collagen metabolism, gave irrefutable proofs to support the inhibition of XKS on Ca^2+^ overloading mechanism in MI (Fig. 6). Importantly, both the tissues-target metabonomics and the western blotting analysis demonstrated that inhibition of Ca^2+^ overloading involved the protection mechanism of XKS against MI metabolic perturbation, which provided a novel molecular insight into understanding the therapeutic action of XKS.

The Ca^2+^ overload induced by excessive *β*-adrenergic agonist (ISO) could initiate the formation of MI, where involves a number of genetic, metabolic, and environmental factors^46^,^47^. Among them, the cell apoptosis by a Ca^2+^-, CaMK II-, and Caspase-3-dependent pathway^24^ is one of the major contributors to the development of MI^48^. Thus, three proteins related with Ca^2+^ overloading mechanism, PLA2 IIA, CaMK II and Procaspase-3, were selected to confirm the inhibition of XKS on the Ca^2+^ overloading mechanism, which could highlight the linking between the upstream protein expression and the downstream endogenous metabolites in molecular pharmacology.

PLA2 IIA is a mediator of systemic inflammation and risk of MI^21^,^22^. Overloading of the intracellular Ca^2+^ can activate the phospholipase (PLA2 IIA) in cell membrane, which promotes the cell membrane disruption^49^. The activation of PLA2 IIA could result in the decreased generation of glycerophospholipids and the elevation of arachidonic acid (**10**)^50^, which contribute to the injury of cellar membrane. In this present work, the over-expression of PLA2 IIA was observed in model group. As a result, the increased arachidonic acid (**10**) and the decreased glycerophospholipids (lysoPC (20:3(8Z,11Z,14Z)) (**3**), lysoPC (18:0) (**5**) and PC (18:4(6Z,9Z,12Z,15Z)/18:0) (**6**)), were observed in rat myocardium with administration of high ISO dose. XKS pretreatment could significantly inhibit the expression of PLA2 IIA and therefore ameliorate the abnormalities of the four metabolites.

CaMK II is a recently identified downstream element of the *β*-AR-initiated signaling cascade that is linked to pathological myocardial remodeling and regulation of the key proteins involved in cardiac excitation-contraction coupling^47^. Activation of CaMK II could initiate several biological factors, such as the opening of mPTP^51^, activation of collagenase and xanthine oxidase^24^,^35^,^52^, and oxidative stress^33^. Furthermore, taurine could regulate the calcium channel, which was associated with the Ca^2+^ overload mechanism^30^. These involved factors initiate the metabolic disturbances of downstream metabolites in molecular pharmacology. In the present work, the over-expression of CaMK II was observed in model group. As a result, the perturbances of four metabolic pathways, *β*-oxidation of fatty acid, collagen metabolism, purine metabolism and glutathi*o*ne metabolism, were observed in MI myocardium. Interestingly, XKS treatment significantly inhibited the up-regulation of CaMK II and the consecutive metabolic disturbances. These results suggested that the regulation of CaMK II might be a prominent target for XKS pretreatment against MI.

Caspase-3 is a key effector enzyme of apoptosis, and its activation depends on the participation of cytosolic free calcium^45^. In our previous study, accumulation of plasma phytosphingosine could result in the opening of the mPTP, subsequent release of Cytochrome C and activation of Caspase-3^53^, which was approved in our study. With treatment of XKS, the over-expression of Pro-Caspase-3 was down-regulated, indicating the cardiomyocyte apoptosis induced by Ca^2+^ overload was inhibited.

Specially, myocardial tissues including cardiac cells, fiber cells, smooth muscle cells and other types of cells. Among them, myocardial cells are the structural and functional basis of myocardial excitability, autorhythmicity, conductivity and contraction. H9c2 cell, derived from embryonic BD1X rat heart tissue, exhibits many of the properties of skeletal muscle in myocardium tissue. The stimulation to H9c2 cells could model the myocardial tissue response to varied environment on the micro level^54^. The cell hypertrophy model induced by ISO can partially characterize the MI pathological change of myocardial ischemia *in vitro*. Here, a metabonomic approach was utilized to reveal the metabolic regulation of XKS on ISO-induced hypertrophy in H9c2 cells. As a result, ten potential biomarkers related to ISO-induced hypertrophy were identified and XKS mediated all of them, primarily involving glycerophospholipid metabolism, sphingolipid metabolism and fatty acid *β*-oxidation pathway. Among them, the disturbance of sphingolipid metabolism was not observed in MI myocardium. These metabolic characteristics were partial in agreement with the myocardium-targeted metabolic alterations in MI.

Interesting, the perturbed lipid pathway was also found in cell pathological model. The cell level of dodecanoic acid (**C1**) was showed similar changes to myocardium samples. Meanwhile, altered glycerophospholipids were also involved into the perturbation of H9c2 cell, which were in agreement with the disorder of myocardial tissue. However, their change were opposite in the two mod, the reason needed further to study. Moreover, the increased phytosphingosine could prevent the opening of mMPT and activation the expression of Caspase-3, which was also proved in myocardium^53^. These results obtained in our study all indicated that the metabolic effects of XKS were likely contributed to inhibition of the metabolic pathology, which also partially confirmed the metabolic perturbations of MI myocardium. Meanwhile, the other metabolic alternations in myocardium tissue might ascribe to other cell types in cardiac tissues.

The Ca^2+^ loading mechanism plays a major role in several biological processes, including disorders of energy metabolism, excessive oxidative stress, inflammation as well as disruption of myocardium structures. The Ca^2+^-, CaMK II-, and Caspase-3-dependent pathway thus arises as potential promising targets for myocardial infarction. Furthermore, increasing studies have also marked CaMK II as a determinant of clinically important heart disease phenotypes, and suggest the inhibition of CaMK II can be a highly selective approach for targeting adverse myocardial remodeling linked to *β*-AR signaling^55^. Calcium antagonist (such as verapamil and diltiazem) has been used in the protection from MI, but their side effects including nausea, asthenia, dizziness, hypotension, cardiac arrhythmia and bradycardia limit their usage in clinical. The future development of Ca^2+^ loading inhibitors will rely on a better understanding of the tissue-specific role of Ca^2+^ loading signaling and identification of biomarkers to select those patients who will benefit the most from these drugs.

The metabolic characteristics from rats and H9c2 cell treated with ISO were all related to the Ca^2+^ loading mechanism, which demonstrated that the Ca^2+^ loading mechanism were one of the important pathological mechanisms in ISO-induced MI and the targeted pathway related to XKS regulation. The western blotting results further confirmed the analysis of metabonomics and MetPA, where the interference of calcium dynamics was considered as a prominent target for XKS pretreatment. Furthermore, numerous studies have found some components in XKS have direct inhibited activities related to the Ca^2+^ loading mechanism in MI.

XKS has been identified 51 constituents in our previous study^9^, and 16 of them have been reported to have various regulative mechanisms against MI (Tables S5 and S6). Those biological activities might owe to their direct effect on cardiac myocytes or other cell types. However, they also have other biological activities on the other organs/tissues, which might be indirect effect on the pathology of MI. For examples, danshensu exert its hepatoprotective activity through regulation of intrahepatic JAK/STAT pathway in CCl_4_-induced hepatic injury^56^. While puerarin has potential protection from neuronal apoptosis by reducing the phosphorylation of p38 and JNK in rat hippocampal neurons culturea^57^. Therefore the metabolic effect of XKS on myocardium might be an integral action on cardiac myocytes and other various organs/tissues.

Interestingly, some of these compounds also show potential direct inhibition of the Ca^2+^ overloading in the formation of MI. For example, danshensu, salvianolic acid A, salvianolic acid B and puerarin can inhibit *L*-type calcium channel current to prevent Ca^2+^ overloading^58^,^59^,^60^,^61^. Additionally, salvianolic acid B could exert beneficial actions on cardiac function in rats with large MI, mainly recovering the normal expressions of SERCA2a and PLB in myocardium, which is associated with Ca^2+^ overloading^62^. The myocardial protection of ginsenoside Rg1during hypoxia/reoxygenation oxidative injury is partially due to its antioxidative effect and intracellular calcium homeostasis^63^. Ginsenoside Rb1 can shorten action potential (AP) duration of ischemic cardiomyocytes and inhibit the opening of calcium channel, which may be one of the critical mechanisms of anti-myocardial ischemia of ginsenoside Rb1^64^. Our docking experiment also showed that these compounds have potential effect on three proteins involved into Ca^2+^ loading mechanism (Table S7). Five of them, salvianolic acid B, pueroside B, puerarin 7-*O*-glucoside, lithosperimic acid and genistein-8-C-apiosyl(1-6)-glucoside, exerted steadily connections with CaMK IIα. Additionally, several constituent had potential link with the other two proteins related to Ca^2+^ overloading mechanism. Among them, salvianolic acid B showed the best affinity with CaMK IIα, which has been reported in other study^60^. The results indicated that these molecules could be viewed as promising compounds for the inhibition of Ca^2+^ overloading mechanism, though their confirmation is further required.

As a conclusion, targeted myocardium metabonomics *in vivo* demonstrated that the inhibition of XKS on Ca^2+^ overloading was a promising targeted pathway against MI. The western blotting results further confirmed this find, where XKS could significant regulate the over-expressions of three related proteins. The purturbence of H9c2 cell metabolome was also partial in agreement with the myocardium. Meanwhile, the molecular docking analysis indirectly demonstrated that the relationships between the active ingredients and the regulative mechanism of XKS against the Ca^2+^ loading in MI. This finding clarified a new pharmacological mechanism of XKS based on a targeted metabonomics *in vivo* and *in vitro*, suggesting XKS as a promising combination therapy (multi-component medicine) for the treatment of MI.

## Conclusion

A myocardium targeted metabonomics using ultra-performance liquid chromatography/quadrupole time-of-flight mass spectrometry and proton nuclear magnetic resonance was firstly applied to delineate the anti-ischemic effect of XKS, a standardized Chinese patent medicine. XKS regulated the abnormalities of all 22 potential biomarkers (**1**–**22**) associated with ISO-induced MI. The results suggested that the therapeutic effect of XKS may involve in regulating the dysfunctions of glycerophospholipid metabolism, arachidonic acid metabolism, fatty acid *β*-oxidation pathway, taurine and hypotaurine metabolism, glutathione metabolism, purine metabolism, collagen metabolism, glycolysis, glycine, serine and threonine metabolism, pantothenate and CoA biosynthesis, arginine and proline metabolism and histidine metabolism. Among them, the first seven metabolic pathways associated with the Ca^2+^ overloading mechanism in pathological process of MI. The expressions of PLA2 IIA, CaMK II and Pro-Caspase-3 in model group were significantly up-regulated compared with the control group. The result also suggested that XKS significantly inhibited the overexpression of those Ca^2+^ overloading-related proteins. The molecular docking analysis also linked its active ingredients with the proteins related to the Ca^2+^ overloading mechanism. And these evidences demonstrate that the inhibition of Ca^2+^ overloading of XKS might ascribe to their combined functions together. Our findings indicated that XKS protected against ISO-induced MI major *via* its inhibited Ca^2+^ overloading effect and regulation of those related metabolic perturbations, which provided novel insights for the therapeutic mechanism of XKS against MI.

## Methods

### Reagents and Materials

Standardized XKS tablets were supplied by a GMP pharmaceutical company, WoHua Pharmaceutical Co, CHN (batch No. 090629). The fingerprinting was performed by HPLC analysis as previously reported^9^,^10^. HPLC-grade acetonitrile were purchased from J.T. Baker (Phillipsburg, NJ, SA). Isoproterenol hydrochloride (ISO), phosphate buffer (PBS), dimethylsulfoxide (DMSO), 3-(4,5-dimethyl-thiazol-zyl)-2,5-diphenyltetrazo-liumbromide (MTT), 0.25% trypsin solution and deuteriumoxide (D_2_O, 99.9%) with 5% TSP were purchased from Sigma-Alorich (St. Louis, USA). The assay kits for lactate dehydrogenase (LDH), creatinenases (CK), aspartate transaminase (AST), superoxide dismutase (SOD) and malondialdehyde (MDA) were purchased from Nanjing Jiancheng Bioengineering Institute (Nanjing, China). Complete medium (CM) composed of Dulbecco’s modified Eagle high glucose medium (DMEM) containing 10% (v/v) fetal bovine serum (FBS) was provided by GIBCO, Inc., Ultrapure water (18.2 MΩ) was prepared with a Milli-Q water purification system (Millipore, France). Antibodies against Phospholipase A2 IIA (PLA2 IIA), calcium/calmodulin-dependent protein kinase II (CaMK II), Pro-Caspase-3 and GAPDH were supplied by Epitomics (Epitomics Inc., USA). All other used chemicals were of analytical grade.

### Animal treatment and sample collection

Wistar rats, male, weighing 196.4 ± 8.2 g from the institute of Laboratory Animal Science, CAMS & PUMC (Beijing, China), were housed individually in cages and maintained (20–25 °C and 40%–60% humidity) under a standard 12 h light/dark cycle with free access to purified water and commercial diet in Specific Pathogen Free Laboratory. All experiments were performed in accordance with the guidelines for the Principles of Laboratory Animal Care and Use of Laboratory Animals published by NIH (NIH Publication, 8th Edition, 2011). All experimental procedures were approved by the Ethics Committee of the Institute of Medicinal Plant Development, CAMS & PUMC. Rats were randomly divided into four groups (n = 6) and equalized with body weights. ISO (85 mg/kg) was dissolved in normal saline and injected subcutaneously to rats at an interval of 24 h at the last two days to induce experimental MI. Group I (control group) received saline, group II (model group) received ISO, group III (positive group) received propranolol (0.10 g/kg for 4 weeks) and ISO, group IV (XKS group) received XKS (2.88 g/kg for 4 weeks) and ISO. After 12 h following the last dose of ISO, rats were sacrificed under anesthetized with urethane (1.3 g/kg). Plasma samples were collected and centrifuged at 4000 rpm for 15 min at 4.0 °C and stored at −80 °C until being tested for clinical chemistry analysis. The myocardial tissues were quickly collected for the metabonomic study and western blotting analysis.

### Plasma biochemistry assays

MI was biochemically diagnosed with changed markers of cardiac injury and its oxidative mechanisms. The cardiac injury was evaluated by measuring plasma CK, LDH and AST activities, and the oxidative stress markers including activities of SOD and MDA in plasma were determined using standard kits (Nanjing Jiancheng Institute of Biotechnology) on a spectrophotometry (Mapada, UV-3100, China) according to the manufacturer’s instruction.

### Pathology

Myocardial tissues from all the groups were subjected to pathological observations. The lower portion of myocardial tissue from each heart was fixed in 10% buffered formalin solution for 48 h, embedded in paraffin, 5 *μ*m sectioned, and stained with hematoxylin-eosin (HE). Images were obtained and studied under light microscopy (Olympus, BX53, Japan).

### Myocardium sample preparation for LC-MS and NMR analyses

A two-step extraction procedure was carried out according to our previous study^17^. For each 50 mg of myocardium sample, 2,000 *μ*L of the chilled extraction solvents (the mixture of chloroform, methanol and water (1:2:1, v/v/v)) were added to the tissue sections in a tube. The samples were homogenized keeping in ice bath with a homogenizer (IKA, Germany) at 30,000 rpm. And then the homogenate was centrifuged at 3,000 rpm for 15 min at 4 °C. A 1,500 *μ*L aliquot of supernatant was transferred into a fresh tube, and then the deposit was re-homogenized with 2,000 *μ*L of chilled methanol. 1,500 *μ*L aliquot of supernatant was transferred to the above tube after centrifugation for drying, and then the dried residue was dissolved as described below. Each sample was prepared in duplicate for both of UPLC-Q/TOF MS and ^1^H NMR analyses.

For UPLC-Q/TOF MS analysis, the dried residue was dissolved in 100 *μ*L of the initial mobile phase, and 2 *μ*L were injected into the UPLC system for analysis after centrifugation for 15 min at 13,000 rpm.

For ^1^H NMR analysis, the dried residue was dissolved in 580 *μ*L of 0.9% NaCl (w/v) solution containing 20% of D_2_O as a field lock. The mixture was centrifuged (13,000 rpm, 10 min) and the supernatant (550 *μ*L) of each sample was then transferred into a 5-mm o.d. NMR tube individually.

### UPLC-Q/TOF MS Analysis of myocardium sample

UPLC-Q/TOF MS analyses were performed on Waters Acquity^TM^ Ultra Performance LC system (Waters Corporation, Milford, MA, USA) equipped with an Acquity UPLC BEH C18 column (2.1 × 100 mm, 1.7 *μ*m, Waters Corp., Milford, USA). The column was maintained at 40 °C and eluted at a flow rate of 0.5 mL/min, using a mobile phase of (A) 0.1% (by volume) formic acid in water and (B) acetonitrile. The gradient program was optimized as follows: 0–0.1min, 10% B; 0.1–4 min, 10% B to 60% B; 4–9 min, 60% B to 99% B; 9–12 min, washing with 99% B and 12–15 min, equilibration with 10% B. The column eluent was directed to the mass spectrometer without split.

Mass spectrometry was performed on a Waters SYNAPY G2 HDMS (Waters Corp., Manchester, UK) with an electrospray ionization source (ESI) operating in positive and negative modes. The capillary voltages were set at 3.0 and 2.5 KV, respectively. The sample cone voltage was set at 40 V and the extraction cone voltage was set at 4.0 V. Nitrogen was used as the drying gas. The desolvation gas rate was set to 800 L/h at a temperature of 450 °C, the cone gas rate was set at 40 L/h, and the source temperature at 120 °C. The scan time and inter scan delay were set to 0.15 and 0.02 s, respectively. All analyses were acquired using a lockspray interface to ensure accuracy and reproducibility at a flow rate of 5 *μ*L/min (*m/z* 556.2771 for positive mode and *m/z* 554.2615 for negative mode). Data was collected in centroid mode from *m/z* 100 to 1500. The lockspray frequency was set at 5 s and the lockmass data were averaged over 10 scans for correction.

The raw MS spectra were first analyzed using MarkerLynx Applications Manager Version 4.1 (Waters Corp., Manchester, UK). And then the processed data was then exported and processed by the principal component analysis (PCA) and orthogonal to partial least-squares-discriminate analysis (OPLS-DA) in the software package Simca-P software (v13.0.3, Umetric, Umeå, Sweden). A typical 7-round cross-validation was applied to validate the model, where the values of *Q*^2^ and *R*^2^ were used to evaluate the models against overfitting. The OPLS-DA analysis is a better and supervised method to pick out discriminating ions contributing to the classification among the experimental samples. All the tested groups were discriminated in the PCA model. In the OPLS-DA model, samples from two groups were classified, and the results were visualized in the form of score plot to show the group clusters and S-plot to show variables contributing to the classification. To search the potential biomarkers, a parameter VIP (Variable Importance in the Projection) was employed to reflect the variable importance. It is considered that the ions with large VIP (VIP > 1) are the most relevant for explaining the classification.

### ^1^H NMR spectroscopic analysis of myocardium sample

All ^1^H NMR spectra were recorded at 300 K on a Bruker AVIII 600 spectrometer (Bruker-Biospin, Germany) equipped with an inverse 5-mm Bruker probe operating at 600.13 MHz ^1^H frequency. ^1^H NMR spectra were acquired using the water-suppressed Carr-Purcell-Mei boom-Gill (CPMG) spin-echo pulse sequence (RD-90°-(τ-180°-τ)n-ACQ). These spectra were measured using a spin-echo loop time (2nτ) of 35 ms to attenuate broad signals from lipids and proteins and only to assist observation of small metabolites. The 90° pulse length was adjusted to 10 *μ*s; a total of 128 transients were collected into 72 K data points over a spectral width of 20 ppm with a recycle delay (RD) of 4 s and an acquisition time of 3.07 s.

Prior to Fourier transformation, the FIDs for one-dimensional data were multiplied by an exponential function equivalent to a line broadening factor of 0.5 Hz and were zero-filled to 128 K. All NMR spectra were then corrected for phase and baseline distortions using Topspin software (v2.1, Bruker-Biospin, Germany) and referenced internally to the chemical shift of the methyl resonance of lactate at δ 1.33. The spectra were divided and signal integral computed in 0.01 ppm intervals across the region δ 0.5–9.5 using AMIX software package (v3.9.2, Bruker-Biospin, Germany). The region δ 4.67–5.10 was removed to avoid the effect of residue water saturation. The data was then normalized to the total sum of the spectra prior to data analysis. Multivariate data analysis and modeling were performed using Simca-P software (v13.0.3, Umetric, Umeå, Sweden). PCA and OPLS-DA analyses were processed as the above report as the same procedure as UPLC-Q/TOF MS.

### Protein extraction and Western blotting analysis of myocardium sample

For Western blotting analysis, the myocardium samples were washed twice with cold PBS, and then lysed in appropriate volume of cold lysis buffer (Beyotime Institute of Biotechnology, Jiangshu, China) contained 0.1 mM PMSF. Lysates were centrifuged at 13, 000 rpm for 20 min at 4 °C. Then, the total protein was obtained, and the protein content was determined by Coomassie brilliant blue G. Western blotting assays were performed as follows: protein (4 mg/mL) was denatured by mixing with an equal volume of 5× sample loading buffer and then boiling at 100 °C for 5 min. An aliquot (20 *μ*L, containing 20 *μ*g protein) of the supernatant was loaded onto a SDS gel, separated electrophoresis, and transferred to a NC membrane. 10% polyacrylamide gel was used for all the electrophoresis. The transmembrane time for PLA2 IIA, CaMKII, Pro-Caspase-3 and GAPDH were all 40 min. After the NC membrane was incubated with 3% BSA-TBST for 30 min, the membrane was incubated with primary antibodies overnight at 4 °C and incubated either rabbit anti-PLA2 IIA (1:2,000 dilution), rabbit anti-CaMKII (1:2,000 dilution), rabbit anti-Pro-Caspase-3 (1:5,000 dilution) and mouse anti-GAPDH (1:20,000 dilution). Blots were then incubated with horse radish peroxidase-conjugated goat anti-rabbit Ig G (Beijing TDY Biotech CO., Ltd., Beijing, China) or horseradish peroxidase-conjugated goat anti-mouse Ig G (Beijing TDY Biotech CO., Ltd., Beijing, China) for 40 min at room temperature at a 1:20,000 dilution. To calculate the fold change, the density of the protein bands was determined using the Image Quant TL software provided by GE.

### H9c2 cell lines and MTT assay

For this experiment, rat fetal cardiomyocytes (H9c2) human hepato were obtained from the Type Culture Collection of the Chinese Academy of Sciences, Shanghai, China. H9c2 cells were grown in CM under humid atmosphere at37 °C in CO_2_ incubator. Cell cultures between passages 3 to 5 were used for each experiment.

The cultured H9c2 cells were detached by trypsinization, centrifuged at 1000 rpm for 5 min and resuspended in fresh CM at a density of 5 × 10^4^ cells/mL. Then, 100 *μ*L of the cells were planted onto 96-well flat bottom plates. After incubation in 5% CO_2_-air mixture at 37 °C for 24 h, the cells were pretreated with the XKS (0.25, 0.125 and 0.0625 mg/mL) and cultured at 37 °C for an additional 24 h, then ISO (15 *μ*M) was added into the plate and cultured at 37 °C for an additional 24 h. and the cancer cell without drugs and ISO was used as control. 20 mL of MTT stock solution (5 mg/mL in PBS without phenol red in the dark and filtered through a 0.2 *μ*m filter before use) was then added into each well. After incubating for 4 h at 37 °C, 150 *μ*L DMSO was added to each well to dissolve the formazan crystals. Then the plates were gently shaken for 1 min and determined by a microplate reader (Thermo, USA) at 490 nm. The morphology of the cells was evaluated by inverted microscope (MQX 200, BioTek, USA).

### H9c2 cell sample collection

The cultured H9c2 cells were detached by trypsinization, centrifuged at 1000 rpm for 5 min and resuspended in fresh CM at a density of 5 × 10^4^ cells/mL. Then, 2 mL of the cells were planted onto 6-well plates. After incubation in 5% CO_2_-air mixture at 37 °C for 24 h, the cells were pretreated with the XKS (0.25 mg/mL) and cultured at 37 °C for an additional 24 h, then ISO (15 *μ*M) was added into the plate and cultured at 37 °C for an additional 24 h. And the cell without drugs and ISO was used as control.

At the end of treatment (n = 6), the media was removed, all cells were washed with cold phosphate buffer saline (PBS) three times immediately before liquid nitrogen was added to each well to quench metabolism. Then cells were harvested using a cell scraper from each well which were added to 2 mL of 80% (v/v) methanol (cooled at −80 °C), and the suspension of the cell in each well was transferred to a 2 mL eppendorf tube. All the cell suspensions were lysed by five cycles of freeze-thaw (fresh frozen in liquid nitrogen for 5 min and thawed at 37 °C for 5 min). And the cell suspensions were added into 500 *μ*L 80% (v/v) cooled methanol to the pellet in a 2 mL eppendorf tube and vortex for 1 min, and centrifuged at 13,000 rpm for 10 min at 4 °C. The cell suspensions were transferred to a new 2 mL eppendorf tube and blow-dried under nitrogen, finally stored at −80 °C until analysis.

### H9c2 cell sample preparation for LC-MS analysis

All samples were thawed at room temperature, and dissolved in 100 *μ*L of the initial mobile phase, and 2 *μ*L were injected into the UPLC system for analysis after centrifugation for 15 min at 13,000 rpm.

### UPLC-Q/TOF MS analysis of H9c2 cell sample

Chromatographic separation was performed on an Acquity UPLC HSS T3 column (2.1 × 100 mm, 1.8 *μ*m, Waters Corp., Milford, USA) using a Waters ACQUITY UPLC system, equipped with a binary solvent delivery system. The column was maintained at 40 °C and eluted at a flowing rate of 0.45 mL/min, using a mobile phase of (A) 0.1% (by volume) formic acid in water and (B) acetonitrile. The gradient program was optimized as follows: 0–1 min, 0.1% B; 1–2 min, 0.1% B to 35% B; 2–4 min, 35% B to 99% B; 9–12 min, washing with 99% B, and 12–15 min, equilibration with 1% B. The eluent from the column was directed to the mass spectrometer without split.

A Waters SYNAPY G2 HDMS (Waters Corp., Manchester, UK) was used to carry out the mass spectrometry with an electrospray ionization source (ESI) operating in positive ion mode. The capillary voltages were set at 3.0 KV in positive mode. Sample cone voltage was set at 40 V. Extraction cone voltage was set at 4.0 V. Used drying gas nitrogen, the desolvation gas rate was set to 800 L/h at 450 °C, the cone gas rate at 40 L/h, and the source temperature at 120 °C. The scan time and inter scan delay were set to 0.15 and 0.02 s, respectively. Leucine-enkephalin was used as the lockmass in all analyses (*m/z* 556.2771 for positive ion mode) at a concentration of 0.5 *μ*g/mL with a flow rate of 5 *μ*L/min. Data was collected in centroid mode from *m/z* 100 to *m/z* 1500. The lock spray frequency was set at 5 s and the lock mass data were averaged over 10 scans for correction.

To ensure the available for the following metabonomic study, a quality control (QC) sample was prepared by pooling the same volume (10 *μ*L) from each cell sample. In the evaluation of system stability, QC sample was analyzed randomly through the experiment to monitor instrument stability in terms of retention time and mass. The repeatability of the method was evaluated using six replicates by analyzing QC sample. As showed in Table S4, the both assessment data acquired from the QC sample indicated that the established method is highly stable and repeatable, and suited for the whole cell metabonomic experiment.

The raw MS spectra were first analyzed using MarkerLynx Applications Manager version 4.1 (Waters Corp., Manchester, UK), which allowed deconvolution, alignment and data reduction to give a list of mass and retention time pairs with corresponding peak area for all the detected peaks from each file in the data set. The main parameters in MarkerLynx were set as follows: retention time range, 0–11 min; mass range, 100–1200 Da; XIC window, 0.02 min; automatically calculate peak width and peak-peak baseline noise; use the raw data during the deconvolution procedure; marker intensity threshold (count), 100; mass tolerance, 0.02 Da; retention time windows, 0.2 min; noise elimination level, 6; retain the isotopic peaks.

### Computer-Aided Molecular Docking Experiments

Molecular docking of the 51 indentified compounds into the active sites of PLA2 IIA, CaMKIIα, Pro-Caspase-3was carried out by the Windows based software package-Molecular Operating Environment (MOE, Version 2008.10, Chemical Computing Group, Canada). The ligands to be docked were constructed in Discovery Studio (DS, Version 2.5.5, Accelrys Software Inc. USA) and the hydrogen atoms were added according to the appropriate protonation states. Since lexible ligand docking was employed, its geometries required only brief optimization using a fast Deriding-like force field (1000 iterations) in DS. During the process of optimization, the element, bond orders, number of bonds, and valence were taken into consideration when the terms of the energy equation were calculated. After geometry optimization, the final conformers of ligands were used as starting point for docking. The X-ray crystallographic structures of PLA2 IIA, CaMK IIα and Pro-Caspase-3 (PDB code: 2VZ6, 1DB4 and 1GFW) were obtained from the Brookhaven Protein Data Bank (http://www.rcsb.org/pdb). Hydrogen atoms were added to the receptor models according to the appropriate protonation states of the ionizable amino acids at pH 7.0 and the valences of the FAD cofactors (oxidized state) and cocrystallized ligands were corrected and hydrogen atoms were added, also according to the appropriate protonation state at pH 7.0. Automated docking was subsequently carried out with the Docking Suit of MOE 2008. Total ligand flexibility was used in this protocol whereby the final ligand conformations were determined by the Monte Carlo conformation search method set to a variable number of trial runs. The docked ligands were further refined using *in situ*-ligand minimization with the Smart Minimizer algorithm. All parameters for the docking runs were set to their default values and ten possible binding solutions were computed for each docked ligand. The best-ranked binding conformation of ligand was determined according to the Dock Score values.

### Statistical analysis

All values were expressed as mean ± S.D. The significance of differences between the means of the treated and un-treated groups has been compared by two-tailed Student’s *t*-test using the Statistical Package for Social Science program (SPSS 16.0, SPSS, Chicago, IL, USA). The significance threshold was set at *p* < 0.05 for this test.

## Additional Information

**How to cite this article**: Liu, Y.-T. *et al*. Standardized Chinese Formula Xin-Ke-Shu inhibits the myocardium Ca^2+^ overloading and metabolic alternations in isoproterenol-induced myocardial infarction rats. *Sci. Rep.* **6**, 30208; doi: 10.1038/srep30208 (2016).

## Supplementary Material

Supplementary Information
