# Supplementary material for: Standardized Chinese Formula Xin-Ke-Shu inhibits the myocardium Ca2+ overloading and metabolic alternations in isoproterenol-induced myocardial infarction rats
Source: Sci Rep. 2016 Jul 26;6:30208. doi: 10.1038/srep30208 (PMC4960537; doi:10.1038/srep30208)
Supplement: Supplementary Information [file srep30208-s1.pdf]

Standardized Chinese Formula Xin-Ke-Shu inhibits the myocardium Ca^{2+} overloading and metabolic alternations in isoproterenol-induced myocardial infarction rats

Yue-tao Liu [a], Chao Zhou [a], Hong-mei Jia [a], Xing Chang [a], Zhong-Mei Zou [a,*]

a. Institute of Medicinal Plant Development, Chinese Academy of Medical Sciences and Peking Union Medical College, Beijing 100193, P. R. China.

* Correspondence should be addressed to zmzou@implad.ac.cn.

Table S1. Potential biomarkers related to XKS regulation on MI detected by UPLC-Q/TOF MS and ^{1}H NMR.

UPLC-Q/TOF MS								NMR				
NO	TR (min)	*m/z*	Selected Ion	Elemental Composition	Metabolites	VIP	Pathway	NO	δ (ppm) and multiplicity	Metabolites	VIP	Pathway
1	0.59	245.1499	$[2M+Na]^+$	$C_5H_9N_3$	Histamine	1.59	histidine metabolism	12	1.33 (d), 4.10 (q)	Threonine	5.40	glycine, serine and threonine,metabolism
2	0.79	242.1005	$[M+Na]^+$	$C_9H_{17}NO_5$	Pantothenic acid	1.79	pantothenate and Co A biosynthesis	13	1.47 (d), 3.77 (q)	Alanine	1.75	glycine, serine and threonine metabolism
3	5.37	546.3482	[M+H]+	$C_{28}H_{52}NO_7P$	LysoPC(20:3(8Z,11Z,14Z))	1.30	glycerophospholipid metabolism	14	3.00 (s),3.93 (s)	Creatine	4.55	arginine and proline metabolism
4	6.60	400.3420	$[M+H]^+$	$C_{23}H_{45}NO_4$	L-Palmitoylcarnitine	3.69	fatty acid *β*-oxidation pathway	15	3.04 (s), 3.95 (s)	Phosphocreatine	2.10	arginine and proline metabolism
5	6.97	524.3715	$[M+H]^+$	$C_{26}H_{54}NO_7P$	**LysoPC(18:0)**	3.80	glycerophospholipid metabolism	7	3.26 (t), 3.42 (t)	Taurine	5.19	taurine and hypotaurine metabolism
6	8.12	782.5681	$[M+H]^+$	$C_{44}H_{80}NO_8P$	PC(18:4(6Z,9Z,12Z,15Z)/18:0)	2.81	glycerophospholipid metabolism	16	3.30 (t), 7.68 (s),	3-Methylhistamine	2.87	histidine metabolism
7	0.53	124.0066	$[M-H]^-$	$C_2H_7NO_3S$	Taurine	4.23	taurine and hypotaurine metabolism	17	3.39 (t), 3.89 (m),	Glucose1-phosphate	2.24	glycolysis
8	0.56	611.1437	$[M-H]^-$	$C_{20}H_{32}N_6O_{12}S_2$	GSSG	1.31	glutathione metabolism	18	3.53(m)	Glycylproline	2.35	collagen Metabolism
9	0.59	267.0725	$[M-H]^-$	$C_{10}H_{12}N_4O_5$	Inosine	5.09	purine metabolism	19	3.55 (s)	Glycine	3.45	glycine, serine and threonine metabolism
10	7.64	303.2319	[M-H]−	$C_{20}H_{32}O_2$	Arachidonic acid	3.37	arachidonic acid metabolism	20	3.90 (m), 4.28 (q)	Xanthosine	2.07	purine metabolism

11	7.79	279.2318	[M-H]−	$C_{18}H_{32}O_2$	Linoelaidic acid	2.47	fatty acid *β*-oxidation pathway	21	4.03 (s)	Creatinine	2.12	arginine and proline metabolism
								22	5.24 (d)	Glucose	1.91	glycolysis

Table S2. Results of ingenuity pathway analysis with MetPA.

	Total	Hits	Raw p	-log(p)	Holm adjust	FDR	Impact
taurine and hypotaurine metabolism	8	1	0.093209	2.3729	1.0	0.70	0.42857
glycolysis	26	2	0.030156	3.5014	1.0	0.61	0.36027
arachidonic acid metabolism	36	1	0.35905	1.0243	1.0	1.00	0.32601
glycine, serine and threonine metabolism	32	3	0.0059139	5.1304	0.5	0.48	0.29197
histidine metabolism	15	1	0.168	1.7838	1.0	0.91	0.22043
galactose metabolism	26	2	0.037921	3.2722	1.0	0.61	0.08389
amino sugar and nucleotide sugar metabolism	37	2	0.071884	2.6327	1.0	0.71	0.08058
primary bile acid biosynthesis	46	2	0.10484	2.2553	1.0	0.71	0.05952
glycerophospholipid metabolism	30	1	0.30916	1.1739	1.0	1.00	0.04444
glutathione metabolism	26	2	0.037921	3.2722	1.0	0.61	0.04294
pantothenate and CoA biosynthesis	15	1	0.168000	1.7838	1.0	0.91	0.02041
arginine and proline metabolism	44	2	0.097187	2.3311	1.0	0.71	0.01198
purine metabolism	68	2	0.197860	1.6202	1.0	0.94	0.00260
cyanoamino acid metabolism	6	1	0.070705	2.6492	1.0	0.71	0.0
methane metabolism	9	1	0.104270	2.2608	1.0	0.71	0.0
nitrogen metabolism	9	1	0.104270	2.2608	1.0	0.71	0.0
pentose and glucuronate interconversions	14	1	0.157680	1.8472	1.0	0.91	0.0
aminoacyl-tRNA biosynthesis	67	2	0.193380	1.6431	1.0	0.94	0.0
fructose and mannose metabolism	19	1	0.208090	1.5698	1.0	0.94	0.0
porphyrin and chlorophyll metabolism	27	1	0.282870	1.2628	1.0	1.00	0.0
fatty acid metabolism	39	1	0.382700	0.96051	1.0	1.00	0.0

Note: Total is the total number of compounds in the pathway; the Hits is the actually matched number from the user uploaded data; the Raw *p* is the original *p* value calculated from the enrichment analysis; the Impact is the pathway impact value calculated from pathway topology analysis.

Table S3. Stability and reproduction of UPLC-Q/TOF MS based on H9c2 cell metabonomics.

NO.	RT (min)	*m/z*	Stability			Repeatability		
			RSD (%)$_{RT}$	RSD (%)$_{m/z}$	RSD (%)$_{p}$	RSD (%)$_{RT}$	RSD (%)$_{m/z}$	RSD (%)$_{p}$
1	1.13	120.0809	0.1460	0.0009	7.1794	0.3631	0.0016	7.8676
2	2.12	279.0930	0.3508	0.0014	11.2473	0.2271	0.0004	10.3978
3	3.01	218.2124	0.5335	0.0007	12.3873	0.3834	0.0013	10.7496
4	4.16	246.2431	0.0738	0.0016	5.3532	0.1265	0.0010	6.4275
5	5.92	318.3014	0.7537	0.0018	8.0833	0.3742	0.0038	8.3325
6	6.72	415.2199	0.1133	0.0005	5.9040	0.2631	0.0005	12.1038
7	7.74	330.3373	0.3159	0.0007	6.7932	0.2882	0.0013	2.6089
8	8.01	365.3525	0.4375	0.0004	12.2947	0.1390	0.0017	13.3475
9	8.63	524.3691	0.2518	0.0010	8.0349	0.2327	0.0009	5.4758
10	8.86	576.3658	0.0925	0.0009	5.8420	0.3262	0.0010	6.3327

Note: The RSD (%)$_{RT}$, RSD (%)$_{m/z}$ and RSD (%)$_{p}$ values representing for the RSD of retention time, *m/z* and peak areas of the selected ions based on UPLC-Q/TOF MS.

Table S4. Potential biomarkers related to isoproterenol-induced hypertrophy in H9c2 cells detected by UPLC-Q/TOF MS.

NO.	Compound	RT (min)	*m/z*	Adduct Ion	Formula	VIP	Pathway
**C1**	dodecanoic acid	3.01	201.1853	$[M+H]^+$	$C_{12}H_{24}O_2$	2.63	fatty acid β-oxidation pathway
**C2**	phytosphingosine	4.92	340.2821	$[M+H]^+$	$C_{18}H_{39}NO_3$	3.77	sphingolipid metabolism
**C3**	sphinganine	6.83	302.3054	$[M+H]^+$	$C_{18}H_{39}NO_2$	11.59	sphingolipid metabolism
**C4**	unknown	5.53	279.0937	$[M+H]^+$	$C_5H_7N_{14}O$	2.87	unknown
**C5**	lysoPC(18:2)	5.76	520.3326	$[M+H]^+$	$C_{27}H_{51}NO_9P$	1.99	glycerophospholipid metabolism
**C6**	dihydroceramide	5.99	330.3006	$[M+H]^+$	$C_{19}H_{39}NO_3$	14.6	sphingolipid metabolism
**C7**	lysoPC(20:4)	7.4	544.3396	$[M+H]^+$	$C_{28}H_{50}NO_7P$	1.69	glycerophospholipid metabolism
**C8**	PC(16:0/0:0)	7.68	496.3396	$[M+H]^+$	$C_{24}H_{50}NO_7P$	2.94	glycerophospholipid metabolism
**C9**	lysoPC(18:1)	7.94	522.3554	$[M+H]^+$	$C_{26}H_{52}NO_7P$	2.35	glycerophospholipid metabolism
**C10**	cer(d18:0/26:0)	8.64	682.1823	$[M+H]^+$	$C_{44}H_{89}NO_3$	3.21	sphingolipid metabolism

Table S5. The active constituents of XKS with special biological activities against MI.

Compound	Sample source	Mechanism of action	References
Danshensu	Isolated rat hearts	Activation of Akt/ERK1/2/Nrf2 signaling	Int J Clin Exp Med 2015, 8:14793-14804
Protocatechualdehyde	MI rat	decreasing infarct size, myocardial apo ptosis and caspase-3 activity	J Ethnopharmacol 2009,121:268-273
Chlorogenic acid	C57BL/6 mice	Attenuating chronic ventricular remodeling	Int Heart J 2013, 54:176-180
Puerarin	Heart tissues	Increasing of VEGFA, Ang-1 and Ang-2	Int J Clin Exp Med 2015, 8:20821-20828
	AB mice	Meditation by the blockade of PI3K/Akt and JNK signaling pathways	J Cardiol 2014, 63:73-81
	Rat heart	induce VEGF and eNOS expression	Biol Pharm Bull 2006, 29:945-950
Daidzin	Rat	Prevention of oxidative-stress	Can J Physiol Pharmacol 2012, 90:1095-1103
Hyperoside	Isolated myocardi al I/R injury	I/R-induced oxidative stress through the activation of ERK-dependent signaling	Free Radic Biol Med 2013, 57:132-140
Pueroside B	Embryonic stem cells	Inhibition of HIF1α, BNIP3, and cleavage caspase 3 expression	Cell Transplan 2015, 24:561-571
	Plasma and Heart tissues	Alleviatating of oxidative stress, reduce calcium overload, improve endothelial function	Asian Pac J Trop Med 2014, 7:280-284
Rosmarinic acid	Serum and myocardium	Antioxidative activities	Daru 2012, 20:87
Notoginsenoside R1	H9c2 cells	Inhibition of ROCK and enhancing mitochondrial ATP synthase δ-subunits	Am J Physiol Heart Circ Physiol 2014, 307:H1764-H1776

Daidzein	Rat hearts and human umbilical vein endothelial cells	Inhibition of NF-kappaB activation	Life Sci 2009, 84:227-234
Ginsenoside Rg1	Rat myocardial sampl es	Activation of PI3K/Akt and inhibition of p38 MAPK	J Mol Med (Berl) 2011, 89:363-375
	Rabbit myocardiac tissues	stimulate the interstitial granulocyte colony-stimulating factor secretion	Zhongguo Zhong Xi Yi Jie He Za Zhi 2005, 25:916-919
Ginsenoside Re	Rat serum	Regulation of the metabolic alternation	Evid Based Complement Alternat Med 2013, 2013:823121.
Salvianolic acid A	Cardiomyocytes	Inhibition of DUSP2-mediated JNK dephosphorylation and activating DUSP4/16-mediated ERK1/2 phosphorylation.	PLoS One 2014, 9:e102292
	ISO-induced MI rat	Attenuation of ISO-induced cardiac dysfunction and myocardial injur y and improved mitochondrial respiratory function	Eur J Pharmacol 2009, 615:125-132
Salvianolic acid B	MI rat	Increasing left ventricle wall thickness, improve heart contractility, and decrease heart fibrosis	BMC Pharmacol 2010, 10:10
	Endothelial progenitor cells and bone mesenchymal stem cells	Regulation of the gene expression of Nkx2.5 and GATA-4 mRNA	Zhongguo Zhong Xi Yi Jie He Za Zhi 2009, 29:529-532
Genistein	Transplanted ECFCs	Enhanced ECFCs' migration and proliferation, which was accompanied by increases in the expression of ILK, α-parvin, F-actin,	PLoS One 2014, 9:e96155

		and phospholylation of ERK 1/2 signaling	
Ginsenoside Rb1	Rat myocardium	Inhibiting p38α MAPK phosphorylation	Can J Physiol Pharmacol 2016, 94:97-103
	Diabetic rats	Activation of PI3 K/Akt pathway	Mol Biol Rep 2011, 38:4327-4335

Table S6. The active constituents of XKS with special biological activities in other experimental models.

Compound	Experimental model	Mechanism of action	References
Danshensu	CCl_4-induced hepatic injury	Regulation of intrahepatic JAK/STAT pathway	Pathol Biol (Paris) 2014, 62:348-353
Protocatechualdehyde	Cerebral Ischemia-Reperfusion-Induced Oxidative Injury	Neuroprotective effect involves the PKCε/Nrf2/HO-1 pathway.	Mol Neurobiol 2016, doi:10.1007/s12035-016-9690-z
Chlorogenic acid	Alcohol-induced damage in PC12 cells	Enhancement of the expression of GAP-43 and the inhibition of mitochondrial apoptotic pathway	Biomed Pharmacother 2016, 79:254-262
Puerarin	Neuronal apoptosis induced by induced by high glucose	reducing the phosphorylation of p38 and JNK	Int J Clin Exp Med 2015, 8:20821-20828
Daidzin	Apoptotic effect in human retinal pigment epithelial (RPE) cells cultured with methylglyoxal		J Ethnopharmacol 2014, 152:594-598
Hyperoside	H_2O_2‑induced oxidative damage on human melanocytes	Regulation of B‑cell lymphoma‑2/Bcl‑2‑associated X protein and Caspase 3	Mol Med Rep 2016, doi: 10.3892/mmr.2016.5107
Pueroside B	Apoptotic effect in human retinal pigment epithelial cells cultured with methylglyoxal		J Ethnopharmacol 2014, 152:594-598
Rosmarinic acid	Spinal cord injury	Downregulation of NF-κB and pro-inflammatory cytokines	Nutr Neurosci 2016, doi:10.1080/1028415X.2015.1103460
Notoginsenoside R1	Amyloid-β (1-42) induced mitochondrial apoptotic death in	Activation of Caspase-3 and caspase-9	Zhongguo Zhong Yao Za Zhi 2015, 40:303-307

	SH-SY5Y cells		
Daidzein	Apoptotic effect in human retinal pigment epithelial (RPE) cells cultured with methylglyoxal		J Ethnopharmacol 2014, 152:594-598
Ginsenoside Rg1	Chronic stress induced depression	Activating the CREB-BDNF system within the prefrontal cortex	Eur J Neurosci.2016, doi: 10.1111/ejn.13255
Ginsenoside Re	Amyotrophic lateral sclerosis	Inhibition the TLR4 pathway	Am J Chin Med 2016, 44:401-413
Salvianolic acid A	Angiotensin-II induced murine peritoneal macrophages	Attenuates cell apoptosis, oxidative stress, Akt and NF-κB activation	Curr Pharm Biotechnol 2016, 17:283-290
Salvianolic acid B	Oligodendrocyte precursor cell differentiation	Protection of axons, myelin sheath and the recovery of neurological function.	Neural Regen Res 2016, 11:487-492
Genistein	Pulmonary arterial hypertension	Activating PI3K/Akt/eNOS signaling	Histol Histopathol. 2016, doi: 10.14670/HH-11-768
Ginsenoside Rb1	Severe cerebral ischemia-induced injuries in aged mice	Inhibition of the involvement of oxidative stress and extracellular signal-regulated kinase activation	Geriatr Gerontol Int 2015, doi:10.1111/ggi.12699

Table S7. The five highest ranked compounds within the tested three protein sites according to analysis of computer-aided molecular docking.

Rank NO.	PLA2		CaMK II α		Pro-Caspase-3	
	Compounds	LibDock Score	Compounds	LibDock Score	Compounds	LibDock Score
1	Salvianolic acid B	198.213	Salvianolic acid B	203.22	Salvianolic acid B	204.846
2	Puerarin 7-*O*-glucoside	178.27	Pueroside B	187.32	Ginsenoside Rb1	174.795
3	Ginsenoside Rg3	174.006	Puerarin 7-O-glucoside	165.173	NotoginsenosideK/ Gypenoside XVII	165.175
4	Salvianolic acid C	169.959	Lithosperimic acid	162.637	Ginsenoside Rd	164.054
5	Lithosperimic acid	167.902	Genistein-8-C-apiosyl(1-6)-glucoside	162.108	Notoginsenoside R1	164.004

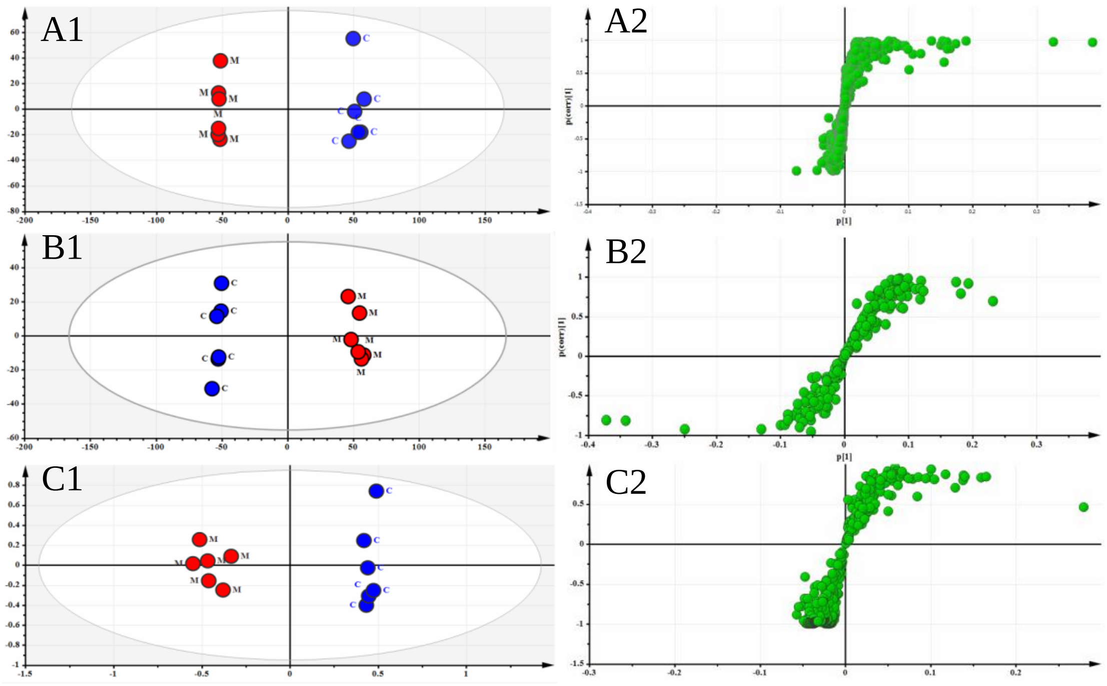

Figure S1. OPLS score plot and S-plot of myocardium samples collected from the control and model groups based on UPLC-Q/TOF MS in positive mode (A1 and A2, R^2X = 0.967, R^2Y = 1, Q^2 (cum)= 0.967) and negative mode (B1 and B2, R^2X = 0.66, R^2Y = 1, Q^2 (cum) = 0.996). OPLS score plot and S-plot of myocardium samples collected from the control and model groups based on ^{1}H NMR (C1 and C2, R^2X = 0.987, R^2Y = 0.999, Q^2 (cum) = 0.874). (C), Control Group, (M), Model Group.

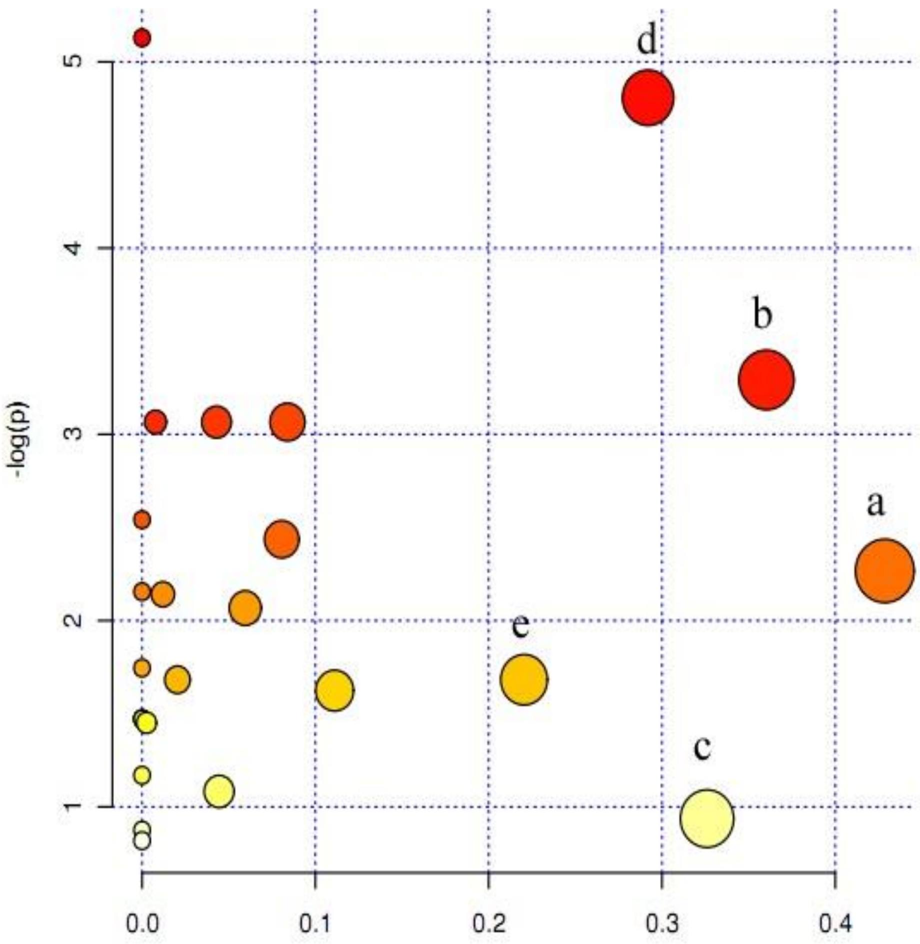

Figure S2. Summary of pathway analysis with MetPA. a, taurine and hypotaurine metabolism; b, glycolysis; c, arachidonic acid metabolism; d, glycine, serine and threonine metabolism; e, histidine metabolism.

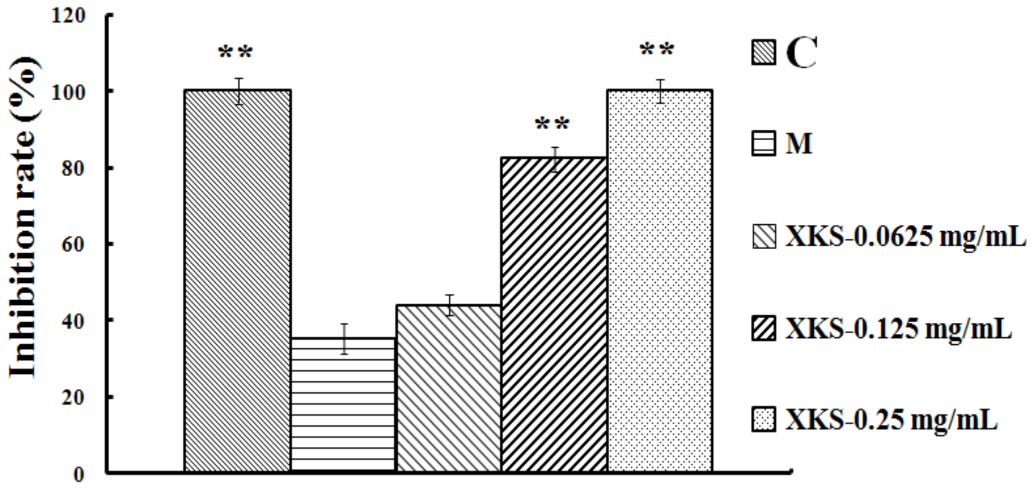

Figure S3. Inhibitory effects of XKS against isoproterenol-induced hypertrophy in H9c2 cells at different concentrations. Compared with model group, * $p<0.05$; ** $p<0.01$.

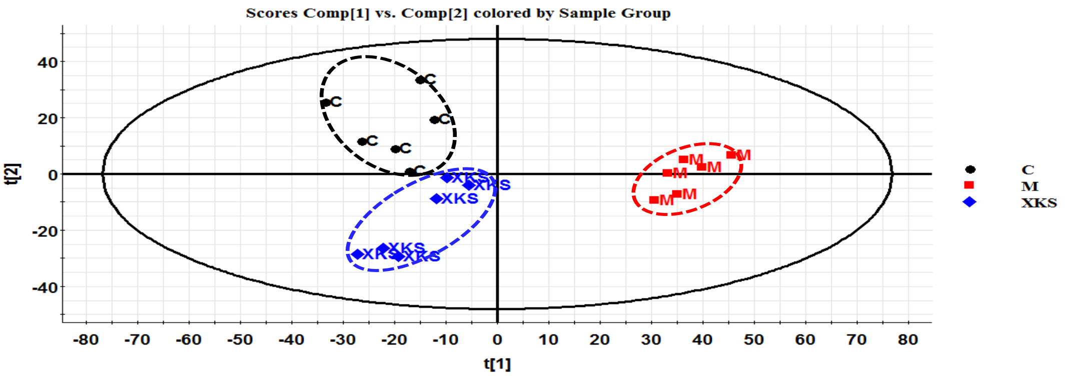

Figure S4. PCA score plot of cell samples collected from different treatment groups based on UPLC-Q/TOF MS data in positive mode. C: Control group, M: Model group, XKS: XKS group.

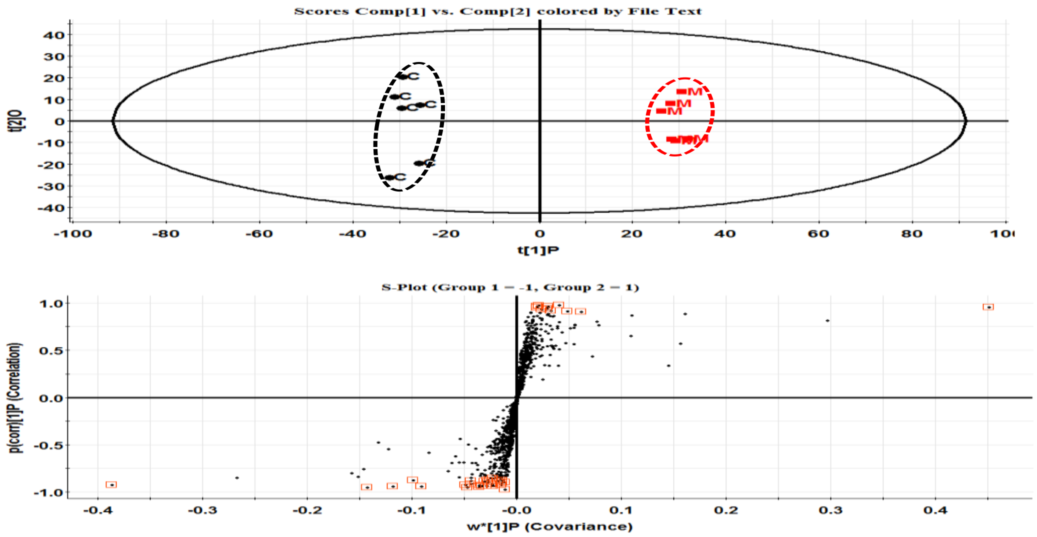

Figure S5. OPLS-DA score plot and S-plot of the control and model groups based on UPLC-Q/TOF MS data in positive mode. C: Control group, M: Model group.

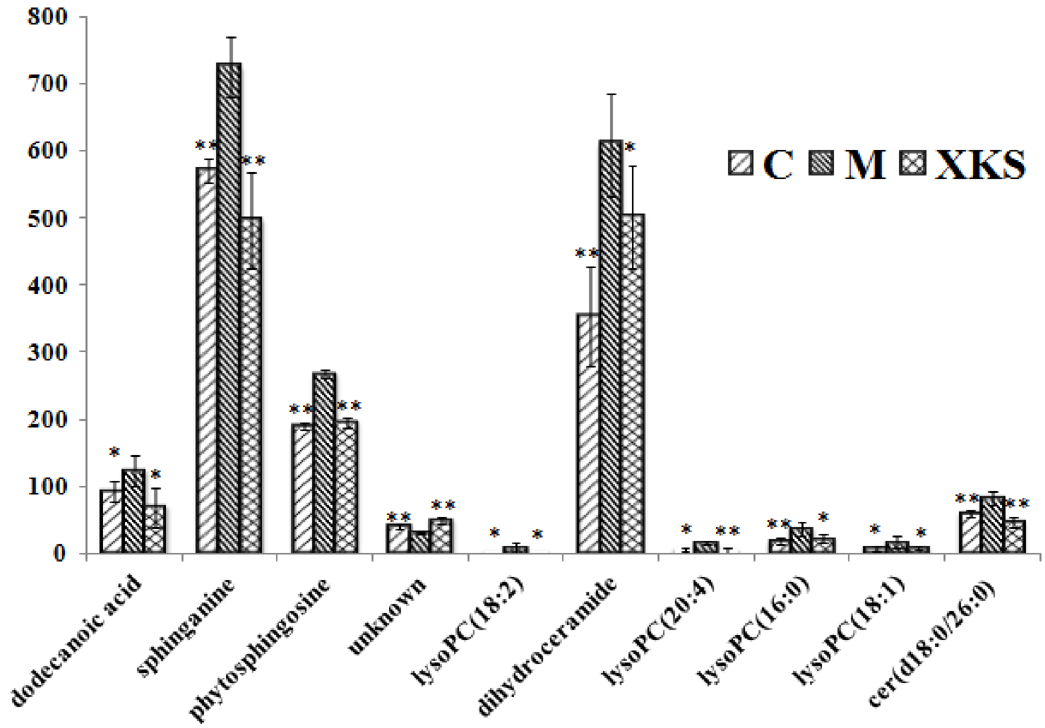

Figure S6. Mean levels of 10 potential biomarkers in H9c2 cells associated with XKS treatment. Compared with model group, * $p < 0.05$; ** $p < 0.01$. C: Control group, M: Model group, XKS: XKS group.
